# Supplementary material for: Omalizumab and Oral Immunotherapy in IgE-Mediated Food Allergy in Children: A Systematic Review and a Meta-Analysis
Source: Pharmaceuticals (Basel). 2025 Mar 20;18(3):437. doi: 10.3390/ph18030437 (PMC11946088; doi:10.3390/ph18030437)
Supplement: Supplementary file 1 [file pharmaceuticals-18-00437-s001.zip › pharmaceuticals-3439275-supplementary.docx]

**PRISMA Checklist for Systematic Review and Meta-Analysis**

**Section 1: Title.** Title: Identifies the study as a systematic review and meta-analysis (Yes/No)

**Section 2: Abstract** 2. Structured summary: Provides an accurate summary of objectives, methods, results, and conclusions (Yes/No)

**Section 3: Introduction** 3. Rationale: Describes the rationale for the systematic review (Yes/No) 4. Objectives: Clearly states the research question and objectives (Yes/No)

**Section 4: Methods** 5. Protocol and registration: Justifies the absence of registration in a public database (Yes/No) 6. Eligibility criteria: Specifies study eligibility criteria (Yes/No) 7. Information sources: Describes all information sources used (Yes/No) 8. Search strategy: Provides the full electronic search strategy (Yes/No) 9. Study selection: Describes the process of study selection (Yes/No) 10. Data collection: Explains how data were extracted and managed (Yes/No) 11. Data items: Lists all variables extracted from studies (Yes/No) 12. Risk of bias: Describes methods for assessing bias (Yes/No) 13. Summary measures: Specifies principal summary measures (Yes/No) 14. Synthesis of results: Explains how results were combined (Yes/No) 15. Additional analyses: Reports any additional analyses performed (Yes/No)

**Section 5: Results** 16. Study selection: Reports the number of studies screened, assessed, and included (Yes/No) 17. Study characteristics: Provides characteristics of included studies (Yes/No) 18. Risk of bias: Summarizes the risk of bias assessments (Yes/No) 19. Results of studies: Presents study findings appropriately (Yes/No) 20. Synthesis of results: Provides a clear synthesis of included studies (Yes/No) 21. Additional analyses: Reports results of additional analyses (Yes/No)

**Section 6: Discussion** 22. Summary of evidence: Summarizes key findings with strengths and limitations (Yes/No) 23. Limitations: Discusses limitations of evidence and methods (Yes/No) 24. Conclusions: Provides a general interpretation of findings (Yes/No)

**Section 7: Funding and Conflicts of Interest** 25. Funding: States sources of funding (Yes/No) 26. Conflicts of Interest: Discloses conflicts of interest (Yes/No)

**Justification for Absence of Registration in Databases:** This systematic review was not registered in a public database such as PROSPERO due to the absence of a predefined protocol at the time of initiation. The primary objective of this review was to provide an up-to-date synthesis of evidence without restricting study inclusion based on a pre-registered framework. Additionally, as PROSPERO typically prioritizes systematic reviews with clinical or interventional components, this study’s focus on observational and meta-analytic data without predefined patient interventions made registration non-essential. Future iterations of this review may consider registration for transparency and methodological rigor.
